# Prevalence and Risk Factors of Confirmed Gestational Diabetes Mellitus Among Pregnant Women With Prior Positive Screening: A Case-Control Study

**DOI:** 10.7759/cureus.61216

**Published:** 2024-05-28

**Authors:** Kholoud Ghamri

**Affiliations:** 1 Internal medicine, King Abdulaziz University Faculty of Medicine, Jeddah, SAU

**Keywords:** rhesus, abo, glucose tolerance test, high-risk pregnancies, risk factors, gestational diabetes mellitus

## Abstract

Background: Saudi Arabia has a higher rate of gestational diabetes mellitus (GDM) than most other countries. There is a paucity of data on the risk factors for GDM, particularly positive screening for diabetes in the initial period of pregnancy.

Objectives: The aim of this study was to determine the prevalence of confirmed GDM in pregnant women who initially screened positive for GDM, as well as to identify its association with age, nationality, and clinical risk factors.

Patients and methods: This case-control study was conducted retrospectively at a tertiary referral center in Jeddah, Saudi Arabia. It included pregnant women who were referred between January 2019 and December 2022 after having tested positive on a 50 g oral glucose tolerance test (OGTT). They subsequently underwent a 75 g or 100 g confirmatory OGTT at our center. The sociodemographic and clinical characteristics of those with confirmed GDM (cases) and those with negative confirmatory OGTT (controls) were compared.

Results: The majority of participants (75.4%) had confirmed GDM. However, there were no significant differences between cases and controls with regard to age, nationality, or clinical or pregnancy-related factors. Of note, the cohort was characterized by high gravidity and high parity, which may indicate susceptibility to GDM.

Conclusion: The study findings support the usefulness of the 50 g OGTT for the screening of pregnant women at high risk for GDM. In addition, high gravidity and parity may also be risk factors for GDM, warranting closer monitoring for GDM and further research in a high-natality population such as that of Saudi Arabia.

## Introduction

Gestational diabetes mellitus (GDM) is defined as hyperglycemia that develops during pregnancy and resolves after birth, representing the most common complication of gestation [1]. Its pathophysiology is believed to be the result of impaired glucose tolerance due to pancreatic β-cell dysfunction on a background of chronic insulin resistance induced by multiple gestational hormones (i.e., mainly human placental lactogen) [2,3]. This leads to chronic maternal hyperglycemia with secondary fetal hyperinsulinism, causing several maternal and fetal complications. Maternal complications associated with GDM include gestational hypertension/preeclampsia, an increased risk of developing diabetes mellitus, and an increased risk of cesarean delivery. On the other hand, fetal complications include macrosomia, neonatal hypoglycemia, hypocalcemia, polycythemia, hyperbilirubinemia, birth trauma, neonatal respiratory distress syndrome, and increased perinatal mortality [3]. Risk factors for GDM include a past history of GDM, maternal age ≥ 40 years, being overweight or obese, lower levels of education, a family history of diabetes, having a macrosomic baby, non-Caucasian race/ethnicity, and cigarette smoking [4,5].

The most accepted and used screening criteria for GDM worldwide are those established by the International Association of Diabetes and Pregnancy Study Group (IADPSG). According to these criteria, the diagnosis of GDM is based on an oral glucose tolerance test (OGTT), which is carried out in the fasting state using 75 g of glucose at 24-28 weeks of pregnancy. The diagnosis of GDM is made if any of the following OGTT cutoff values are met: glycemia ≥ 92 mg/dL (≥ 5.2 mmol/L) immediately; ≥ 180 mg/dL (≥ 10 mmol/L) after one hour; or ≥ 153 mg/dL (≥ 8.5 mmol/L) after two hours [6]. Once the diagnosis is confirmed, management aims to prevent maternal and fetal complications linked to GDM by normalizing glycemic homeostasis and avoiding excess weight gain during pregnancy [7]. To achieve these goals, patients are initially managed by diet and lifestyle measures, in combination with glucose monitoring. In case of non-improvement with first-line treatment, pharmacological therapy based on insulin or oral hypoglycemic agents [i.e., metformin and glyburide] is initiated [3].

Using the IADPSG diagnostic criteria, a recent meta-analysis conducted by Saeedi et al. estimated the global prevalence of GDM as 14.7% [8]. In Saudi Arabia, compared to the international data, high rates of women diagnosed with GDM are reported [9]. Local studies conducted over the past decade have reported high GDM prevalence rates, reaching up to 51%, with an increasing trend over time [10-12].

Despite extensive research on GDM risk factors, the specific risk profile of pregnant women who have previously screened positive for GDM remains underexplored. This subgroup represents a significant high-risk population, particularly in social and cultural contexts where adherence to pregnancy follow-up and GDM management protocols may be affected. Specifically, women with abnormal 50 g OGTT results in the range of 7.8-11 mmol/L, which do not reach the GDM confirmation threshold of 11.1 mmol/L, may fail to undergo the necessary confirmatory testing. This lack of follow-up can significantly impact the timely management and treatment of GDM.

For this purpose, we conducted this study at a tertiary referral center in Jeddah, Saudi Arabia, to determine the actual risk of GDM in pregnant women who had previously screened positive for GDM using a 50 g OGTT. We also analyzed the associated sociodemographic and clinical factors.

Providing such data has important implications for both clinical practice and public health. Clinically, it highlights the need for rigorous post-screening follow-up to confirm GDM diagnoses and implement timely management strategies. From a public health perspective, identifying the sociodemographic and clinical factors associated with confirmed GDM can inform resource prioritization and targeted educational and awareness programs aimed at improving adherence to management protocols and enhancing maternal and fetal health outcomes.

## Materials and methods

Design

This study used a retrospective case-control design, implemented within the Gynecology and Obstetrics Department of King Abdulaziz University Hospital (KAUH), a tertiary referral center in Jeddah, Saudi Arabia. The study protocol was reviewed and approved by the Ethical Committee of KAUH (#629-23).

Participants

All pregnant women referred between January 2019 and December 2022 due to suspected GDM were considered for the study. Suspected GDM was defined based on a screening test using a 50 g OGTT result of 7.8-11 mmol/L, indicating the need for confirmatory testing with either a 75 g or 100 g OGTT. Patients with a normal 50 g OGTT result (<7.8 mmol/L) and those with results of 11.1 mmol/L or higher (confirmed GDM) were excluded. Women who underwent screening other than the 50 g OGTT (such as fasting or random blood glucose) or those with already confirmed GDM (including those with a 50 g OGTT result of 11.1 mmol/L or higher) at another institution were excluded. Additionally, women with pre-existing diabetes mellitus, those receiving long-term corticosteroid therapy, and those with molar pregnancy were excluded to eliminate confounding factors. However, women with twin or multiple pregnancies were included.

Outcome definition

As part of routine practice, all women with suspected GDM underwent either a 75 g or 100 g OGTT (confirmatory OGTT) to confirm or rule out the presence of GDM. The 100 g OGTT defined GDM if two out of four values exceeded the target: fasting blood glucose level > 95 mg/dL (5.3 mmol/L), 1 hour > 180 mg/dL (10 mmol/L), 2 hours > 155 mg/dL (8.6 mmol/L), and 3 hours > 140 mg/dL (7.8 mmol/L). The 75 g OGTT confirmed GDM if one value exceeded the target: fasting ≥ 5.1 mmol/L, 1 hour ≥ 10 mmol/L, and 2 hours ≥ 8.5 mmol/L. The choice of confirmatory test used was based on the physician's preference and the availability of the test kits. The diagnosis was made at 24-28 weeks of gestation. Participants were then categorized into two groups: those with confirmed GDM (cases) and those with negative confirmatory OGTT (controls). The two groups were compared based on the available demographic and clinical parameters.

Data collection

The data for this study were collected by reviewing the KAUH medical records of the eligible participants. Demographic variables included age and nationality, while clinical data encompassed factors such as blood group, Rhesus status, and past medical history including specific conditions such as heart disease, kidney disease, diabetes, hypertension, obesity, thyroid disease, and other unspecified diseases. Obstetrical history and current pregnancy data, including gravidity, parity, and incidence of conditions such as preeclampsia or gestational hypertension, urinary tract infections (UTIs), nausea or vomiting, and other pregnancy-related factors, were also considered in the comparative analysis.

Statistical methods

Statistical analyses were conducted using the IBM SPSS Statistics for Windows, Version 21 (Released 2012; IBM Corp., Armonk, New York). Descriptive statistics were computed for all study variables. Categorical variables were summarized using frequencies and percentages. For continuous variables such as age, mean and standard deviation (SD) values were calculated. The chi-square test was used to analyze the association between categorical variables in the GDM and non-GDM groups, while the independent samples t-test was used for the comparison of continuous variables between the two groups. For 2×2 tables where at least one cell size was ≤5, Fisher’s exact test was employed instead of the chi-square test. The threshold for statistical significance was set at p <0.05.

## Results

The participants (n=280) had a mean age of 30.61 years (SD=5.54) and were predominantly of Saudi nationality (83.9%). Blood group distribution was led by A (42.9%) and O (41.1%), with the vast majority Rh positive (91.1%). Concerning past medical history, a substantial proportion reported no prior conditions (60.4%), whereas the others had at least one ailment, notably diabetes (21.1%) and hypertension (7.1%). Heart disease, kidney disease, and obesity were rarely reported, each <2% (Table 1).

**Table 1:** Demographic and baseline clinical data (N=280) *Values are presented in mean and standard deviation (SD); all other values are frequencies and percentages, except where otherwise specified.

Parameter	Level	Mean/frequency	SD/percentage
Age^*^	Years	30.61	5.54
	Median, range	31	18–43
Nationality	Saudi Arabia	235	83.9
	Other	45	16.1
Blood group	A	120	42.9
	B	34	12.1
	AB	11	3.9
	O	115	41.1
Rhesus status	Positive	255	91.1
	Negative	25	8.9
Any comorbidity	No	169	60.4
	Yes	111	39.6
Heart disease	No	279	99.6
	Yes	1	0.4
Kidney disease	No	278	99.3
	Yes	2	0.7
Family history of diabetes	No	221	78.9
	Yes	59	21.1
Hypertension	No	260	92.9
	Yes	20	7.1
Obesity	No	277	98.9
	Yes	3	1.1
Thyroid disease	No	264	94.3
	Yes	16	5.7
Other diseases	No	232	82.9
	Yes	48	17.1
Number of chronic diseases	0	169	60.4
	1	77	27.5
	2	30	10.7
	3	4	1.4

In terms of obstetrical history and data from the current pregnancy, the distribution of gravida and parity varied, with a higher prevalence observed for higher gravidity (28.6% for 6+) and a peak parity of 2 (21.1%). Pre-eclampsia or gestational hypertension was reported in a small fraction of participants (5.7%), while UTI and nausea or vomiting were extremely rare, each reported by less than 1% of participants. Other conditions were present in 7.1% of cases. Among the total participants, 211 were confirmed GDM; hence, the prevalence of GDM was estimated at 75.4% (95% CI: 69.9-80.3%; Table 2).

**Table 2:** Obstetrical history and current pregnancy data of the cohort (N=280) *These include minor symptoms such as constipation, edema, etc.

Parameter	Level	Frequency	Percentage
Gravidity	0	2	0.7
	1	34	12.1
	2	43	15.4
	3	45	16.1
	4	44	15.7
	5	32	11.4
	6+	80	28.6
Parity	0	12	4.3
	1	48	17.1
	2	59	21.1
	3	45	16.1
	4	44	15.7
	5	30	10.7
	6+	42	15.0
Preeclampsia or gestational hypertension	No	264	94.3
	Yes	16	5.7
Urinary tract infection during visit	No	279	99.6
	Yes	1	0.4
Nausea or vomiting during visit	No	278	99.3
	Yes	2	0.7
Other symptoms/complaints*	No	260	92.9
	Yes	20	7.1
Gestational diabetes in previous pregnancies	No	69	24.6
	Yes	211	75.4

Analysis of demographic and baseline clinical factors revealed no statistically significant differences between women with GDM (n=211) and those without GDM (n=69) in terms of nationality, blood group, Rh status, past medical history, heart disease, kidney disease, diabetes, hypertension, obesity, thyroid disease, other diseases, and a number of past medical conditions (p > 0.05 for all). Specifically, both groups exhibited similar proportions of participants with no past medical history (approximately 60%). Furthermore, the mean age in both groups was statistically comparable (GDM: 30.45 ± 5.44 years, No GDM: 31.09 ± 5.83 years; p = 0.408). Hence, within this high-risk cohort, these parameters did not differentiate between the GDM and non-GDM cases (Table 3).

**Table 3:** Demographic and baseline clinical factors of gestational diabetes mellitus (GDM) among pregnant women with previous positive screening (N=280) *Values are means and standard deviations, and comparisons were made using the independent t-test. Otherwise, values are frequencies and percentages, and the chi-square or Fisher’s exact test was used for comparison. Percentages are calculated based on the column categories.

Factors	Level	No GDM (N=69)		GDM (N=211)		p-value
Age*	Years	31.09	5.83	30.45	5.44	0.408
Nationality	Saudi Arabia	56	81.2	179	84.8	0.471
	Other	13	18.8	32	15.2	
Blood group	A	34	49.3	86	40.8	0.558
	B	9	13.0	25	11.8	
	AB	2	2.9	9	4.3	
	O	24	34.8	91	43.1	
Rhesus status	Positive	62	89.9	193	91.5	0.683
	Negative	7	10.1	18	8.5	
Any comorbidity	No	42	60.9	127	60.2	0.920
	Yes	27	39.1	84	39.8	
Heart disease	No	69	100.0	210	99.5	0.567
	Yes	0	0.0	1	0.5	
Kidney disease	No	69	100.0	209	99.1	0.417
	Yes	0	0.0	2	0.9	
Family history of diabetes	No	54	78.3	167	79.1	0.876
	Yes	15	21.7	44	20.9	
Hypertension	No	64	92.8	196	92.9	0.969
	Yes	5	7.2	15	7.1	
Obesity	No	68	98.6	209	99.1	0.725
	Yes	1	1.4	2	0.9	
Thyroid disease	No	67	97.1	197	93.4	0.246
	Yes	2	2.9	14	6.6	
Other diseases	No	57	82.6	175	82.9	0.950
	Yes	12	17.4	36	17.1	
Number of chronic diseases	0	42	60.9	127	60.2	0.987
	1	19	27.5	58	27.5	
	2+	8	11.6	26	12.3	

Comparative analysis of obstetrical and pregnancy-related factors among a high-risk population of pregnant women (n=280) exhibited no statistically significant differences between those with GDM (n=211) and without GDM (n=69) in gravida, parity, incidence of preeclampsia or gestational hypertension, UTI, and nausea or vomiting (p>0.05 for all). However, the occurrence of other unspecified conditions during pregnancy was significantly higher in the non-GDM group (13.0%) compared to the GDM group (5.2%; p=0.028), representing the singular significant differentiator within these parameters in this high-risk cohort (Table 4).

**Table 4:** Obstetrical and pregnancy-related factors of gestational diabetes mellitus (GDM) among pregnant women with previous positive screening (N=280) Values are presented as frequencies and percentages, and chi-square or Fisher’s exact test was used for comparison. Percentages are calculated based on the column categories. * Statistically significant result (p<0.05).

Factors	Level	No GDM (N=69)		GDM (N=211)		p-value
Gravidity	0	1	1.4	1	0.5	0.963
	1	7	10.1	27	12.8	
	2	10	14.5	33	15.6	
	3	11	15.9	34	16.1	
	4	12	17.4	32	15.2	
	5	9	13.0	23	10.9	
	6+	19	27.5	61	28.9	
Parity	0	2	2.9	10	4.7	0.177
	1	11	15.9	37	17.5	
	2	15	21.7	44	20.9	
	3	12	17.4	33	15.6	
	4	17	24.6	27	12.8	
	5	3	4.3	27	12.8	
	6+	9	13.0	33	15.6	
Preeclampsia or gestational hypertension	No	66	95.7	198	93.8	0.573
	Yes	3	4.3	13	6.2	
Urinary tract infection during visit	No	69	100.0	210	99.5	0.567
	Yes	0	0.0	1	0.5	
Nausea or vomiting during visit	No	69	100.0	209	99.1	0.417
	Yes	0	0.0	2	0.9	
Other symptoms or complaints	No	60	87.0	200	94.8	0.028*
	Yes	9	13.0	11	5.2	

## Discussion

Summary of the findings

This case-control study was conducted on pregnant patients suspected of GDM, primarily aiming to confirm GDM status and identify associated factors. The majority of the study group had gestational diabetes (75.4%), elucidating the high-risk nature of this cohort. Moreover, 40% of the participants had a gravida history of five or more pregnancies, while 26% had five or more parities. By contrast, lower frequencies were reported for other pregnancy-related conditions, including pre-eclampsia or gestational hypertension (5.7%), UTI, and nausea or vomiting (<1%), and other conditions (7.1%). Notably, there was no association between demographic and baseline clinical factors and the development of GDM (p > 0.05). However, pregnant women without GDM had a significantly higher risk for other unspecified conditions compared to those with GDM (p = 0.028).

High prevalence of GDM among patients with abnormal 50 g GTT

In this study, the prevalence of GDM was 75.4%, suggesting an elevated likelihood of GDM diagnosis in pregnant Saudi women with an initial abnormal 50 g OGTT result, using a subsequent positive 75 g or 100 g OGTT. Mathematically, the established 75.4% prevalence in positively screened individuals corresponds to the positive predictive value (PPV) of the screening test, assuming the inclusion of all positively screened but non-confirmed individuals using the 50 g OGTT. Consistent with these findings, research examining 50 g OGTT screening values reported a 68.8% PPV using the National Diabetes Data Group (NDDG) criteria and 80% with Carpenter-Coustan (CC) criteria for GDM diagnosis [13]. Another study indicated a relatively higher specificity of 50 g OGTT, estimated at 72% for a cutoff plasma glucose value of 130 mg/dL, increasing to 85% for a cutoff at 140 mg/dL [14]. The literature further supports the usefulness and cost-effectiveness of the 50 g OGTT in screening for GDM [15,16]. A systematic review of 19 studies further substantiated these conclusions, estimating a pooled sensitivity of 0.79 and a pooled specificity of 0.74 for the 50 g OGTT [17]. Together, these findings validate the use of the 50 g OGTT for identifying women at high risk for GDM, including those in Saudi Arabia. However, the requirement for confirmatory testing remains an issue for effective management and follow-up.

High gravidity is common among populations at high risk for GDM 

In our study, 40% of participants experienced a minimum of five pregnancies, with 26% having at least five childbirths, implying high gravidity and parity among women at an elevated risk of GDM. Specifically, women with three or more pregnancies are 1.27 times more likely to develop GDM, with the odds increasing to 1.32-fold for women aged 30 or older with two or more pregnancies [18]. Correspondingly, multiparity, a known risk factor for GDM, shows a steady increase in incidence from 3.5% in nulliparous women to 14.6% in women having given birth to four or more children [19]. This increased risk can be attributed to the higher likelihood of obesity in women with multiple pregnancies [20], which subsequently heightens GDM susceptibility by escalating metabolic risk factors [21].

No association between GDM and clinical history

Our study demonstrated no statistical associations between the diagnosis of GDM and the tested demographic and clinical factors at baseline, including age, nationality, blood group, past medical history, heart disease, kidney disease, diabetes, hypertension, obesity, thyroid disease, other diseases, and the number of past medical conditions (p > 0.05 for all). Similarly, Bahkali et al. also failed to identify any correlation between blood group and the development of GDM in a cohort from Saudi Arabia [22]. Additionally, a recent study conducted on 2849 pregnant Chinese women showed no significant associations between thyroid-stimulating hormone concentration, thyroid dysfunction subtypes, thyroid peroxidase antibody positivity, and the risk of GDM occurrence [23]. Moreover, a meta-analysis of 23 studies revealed that GDM has no overall association with chronic kidney disease risk, particularly in white women [24]. However, our other results regarding clinical factors not associated with GDM were inconsistent with the results of previous studies. For example, a strong positive correlation was previously reported between GDM risk and maternal age [25]. Furthermore, patients with GDM were shown to exhibit an increased risk for cardiovascular diseases such as ischemic heart disease, hypertension, and type 2 diabetes, compared with those without GDM [26-28]. It is worth mentioning that most of the studies that showed a significant risk of cardiovascular events among GDM patients examined the occurrence of these events after a GDM pregnancy, while our study tested the correlation between GDM and the already diagnosed cardiovascular conditions, which may explain the apparent discrepancies.

Implications for interventions 

Obstetrics specialists in Saudi Arabia should consider the high probability of GDM in patients with abnormal 50 g OGTT results during early screening. Therefore, they must prioritize stricter follow-up and education of these patients, ensuring that confirmatory tests are conducted at the appropriate time and that adequate management measures are initiated in the case of a positive test. Furthermore, it is necessary to enhance the prevention strategies for GDM in at-risk populations, such as those with high gravidity/parity. Strategies include educating women prior to gestation to increase their physical activity, improve their diet, manage their weight, and cease smoking, because these lifestyle changes have been shown to reduce the rates of GDM [29].

Limitations

The single-center design of the present study inherently introduces selection bias, as the collected data reflect the patient population of a single referral center. These centers typically serve more complicated cases or patients with high-risk profiles, leading to a skewed representation of the overall population. This overrepresentation can exaggerate the prevalence and association of risk factors, thus making the findings less generalizable to other populations or healthcare settings. Moreover, local healthcare practices, patient demographics, and other socio-economic factors specific to a center's locality can further limit the applicability of the results to other settings. For a more comprehensive understanding of the phenomenon investigated, multi-center or population-based studies would be beneficial, as they cover a more diverse and representative patient population, thereby enhancing the generalizability of the findings. Another limitation is the lack of additional data on risk factors, particularly glycemic profiles and 50 g OGTT screening results. This limitation is inherent to the retrospective design, as most patients were screened and referred from other institutions. Analyzing these data would have provided further insights into characterizing the risk profile.

## Conclusions

GDM was confirmed in 75.4% of women who tested positive in screening, validating the efficacy of the 50 g OGTT for GDM screening. This underscores the need for rigorous follow-up and timely management for those who previously tested positive. The observed high gravidity and parity in the cohort suggest an additional risk factor for GDM, emphasizing the importance of targeted screening and monitoring in this population. Future comprehensive investigations in Saudi Arabia are needed to identify modifiable factors affecting both GDM risk and follow-up adherence. A holistic approach that considers all risk factors will inform resource allocation, education, and awareness strategies to enhance the effectiveness of GDM screening and management and improve maternal and fetal outcomes.
